# Clinical evaluation of the use of an mhealth intervention on quality of care provided by Community Health Workers in southwest Niger

**DOI:** 10.7189/jogh.09.010812

**Published:** 2019-06

**Authors:** David Zakus, Moise Moussa, Mahamane Ezechiel, Joannes Paulus Yimbesalu, Patsy Orkar, Caroline Damecour, Annette E Ghee, Matthew MacFarlane, Grace Nganga

**Affiliations:** 1 University of Toronto, Toronto, Canada; 2 Ministry of Public Health, Niamey, Niger; 3 World Vision International, Niamey, Niger; 4 York University, Toronto, Canada; 5 World Vision Canada, Mississauga, Canada; 6 Independent Consultant, Aurora, Canada; 7 World Vision International, London, UK

## Abstract

**Background:**

Under the World Health Organization’s (WHO) integrated community case management (iCCM) Rapid Access Expansion Program (RAcE), World Vision Niger and Canada supported the Niger Ministry of Public Health to implement iCCM in four health districts in Niger in 2013. Community health workers (CHWs), known as *Relais Communautaire* (RCom), were deployed in their communities to diagnose and treat children under five years of age presenting with diarrhea, malaria and pneumonia and refer children with severe illness to the higher-level facilities. Two of the districts in southwest Niger piloted RCom using smartphones equipped with an application to support quality case management and provide good timely clinical data. A two-arm cluster randomized trial assessed the impact of use of the mHealth application mainly on quality of care (QoC), but also on motivation, retention and supervision.

**Methods:**

A two-arm cluster randomized trial was conducted from March to October 2016 in Dosso and Doutchi districts. The intervention arm comprised 66 RCom equipped with a smartphone and 64 in the paper-based control arm. Trained expert clinicians observed each RCom assessing sick children presenting to them (264 in intervention group; 256 in control group), re-assessed each child on the same set of parameters, and made further observations regarding perceptions of motivation, retention, supervision, drug management and caregiver satisfaction. The primary outcome was a QoC score composed of diagnostic and treatment variables. Other factors were assessed by questionnaires.

**Results:**

On average, the mHealth equipped RCom showed a 3.4% higher QoC score (mean difference of 0.83 points). They were more likely to ask about the main danger signs: convulsions (69.7% vs 50.4%, *P* < 0.001); incapacity to drink or eat (79.2% vs 59.4%, *P* < 0.001); vomiting (81.4% vs 69.9%, *P* < 0.01); and lethargy or unconsciousness (92.4% vs 84.8%, *P* < 0.01). Specifically, they consistently asked one more screening question. They were also significantly better at examining for swelling feet (40.2% vs 13.3%, *P* < 0.01) and advising caretakers on diarrhea, drug dosage and administration, and performed (though non-significantly) better when examining cough and breathing rates, referring all conditions, getting children to take prescribed treatments immediately and having caregivers understand treatment continuation. The control group was significantly better at diagnosing fast breathing, bloody diarrhea and severe acute malnutrition; and was somewhat better (non-significant) at treating fever and malaria. With treatment in general of the three diseases, there was no significant difference between the groups. On further inspection, 83% of the intervention group had a QoC score greater than 80% (25 out of 31), whereas only 67% of the control group had comparable performance. With respect to referrals, the intervention group performed better, mostly based on their better assessment of danger signs, with more correct (85% vs 29%) and fewer missed, plus a lower proportion of incorrect referrals, with the reverse being true for the controls (*P* = 0.012). There were no statistically significant differences in motivation, retention and supervision between the two groups, yet intervention RCom reported double the rate of no supervision in the last three months (31.8% vs 15.6%).

**Conclusions:**

Results suggest that use of the mHealth application led to modestly improved QoC through better assessment of the sick children and better referral decisions by RCom, but not to improvement in the actual treatment of malaria, pneumonia and diarrhea. Considering mHealth’s additional costs and logistics, questions around its viability remain. Further implementation could be improved by investing in RCom capacity building, building organization culture and strengthened supervision, all essential areas for improving any CHW program. In this real-world setting, in poor and remote communities in rural Niger, this study did not support the overall value of the mHealth intervention. Much was learned for any future mHealth interventions and scale-up.

Niger, a large arid landlocked country in West Africa on the southern edge of the Sahara Desert, is one of the poorest and least developed in the world, ranking 187 of 188 countries on the United Nations’ Human Development Index [1]. Its economy is mainly dependent on subsistence agriculture and livestock and suffers from threats of frequent droughts, insurgency and displaced people. It has the highest global fertility rate of 7.2 children per woman and equally high rates of infant and child disease and death [2]. In 2016, Niger’s under-five mortality rate was 129 deaths per 1000 live births, or 121 225 deaths of children under age five [3]. Three of the most prevalent childhood illnesses – malaria, pneumonia and diarrhea – account for over 60 per cent of deaths in Nigerien children aged 2 to 59 months [4].

The Integrated Community Case Management (iCCM) strategy, based on the deployment of local community health workers (CHWs), aims to increase access to effective case management for children under five years of age suffering from malaria, pneumonia and diarrhea, by bringing services closer to people living in the hardest-to-reach communities and among the most vulnerable populations [5]. Studies demonstrate that iCCM, in general, is highly cost-effective if well utilized and it has been widely used as a major public health strategy for early diagnosis, treatment and referral of preventable childhood illnesses, especially in malaria-endemic countries in sub-Saharan Africa [6].

Globally, the first implementation of iCCM and similar programs was encouraging. In Ghana, 92% of carers of sick children sought treatment from CHWs trained to manage pneumonia and malaria; 77% sought care within 24 hours of onset. In Zambia, a study of iCCM for pneumonia and malaria found 68% of children with pneumonia received early and appropriate treatment from CHWs, and overtreatment of malaria significantly declined [7]. This was also the case with Ethiopia’s revolutionary Health Extension Worker Program, whereby CHWs became actual employees of the state health system. Similarly, in countries like South Africa, Nigeria, Malawi and Rwanda, national programs integrate CHWs into health care systems and provide sustainable career paths [8].

Studies on CHW performance have demonstrated that they had the necessary knowledge and skills to successfully carry out treatment of childhood diseases [9-14], including the use of rapid diagnostic tests (RDTs) and timers in the assessment and management of malaria and pneumonia, respectively, in children [15], and in the delivery of immunization [16]. Others have highlighted challenges CHWs encounter with less education [17] and with diagnosis in the absence of proper supervision and follow-up [18,19]. Training CHWs but not providing adequate supervision has been found to be a key bottleneck in effective iCCM implementation, motivation and retention [20].

Increased phone use by over 420 million unique mobile subscribers in sub-Saharan Africa today, accounting for 43% penetration rate, has greatly revolutionized the health sector [21]. There is an emerging body of evidence demonstrating how use of mobile phones in health programming – mobile Health or mHealth – improves and reduces the cost of patient monitoring, medication adherence, and health care worker communication, especially in rural areas.

In Malawi, mHealth applications were designed to increase CHW access to health information, decision-making and logistical support. Research, using a mixed-method approach, provided understanding of the frequency of use of text messaging (SMS), the reasons for use and CHW feedback on the quality of medical care and disease management [22]. Promising mHealth interventions have also been developed around medication adherence for HIV/AIDS and tuberculosis (TB) patients; including the SIMpill, a pill dispensing system with an SIM card embedded in a small bottle. Each time the bottle is opened, an SMS text is sent to a central server uniquely linked to the patient’s mobile number. As a result, drug adherence increased by between 86 to 92% with a treatment success rate of 94% within the ten months of SIMpill use. In addition to improving treatment adherence, the SIMpill freed up time for CHWs from their routine daily observations of patients taking their medications [23]. In Kenya, a mobile directly observed therapy model, using video and text messaging to enhance medication adherence for TB patients in rural settings, was preferred by 73% of users [24]. In another study in Rwanda, CHWs used RapidSMS, a mobile phone-based system designed to track pregnancy and reduce existing bottlenecks associated with maternal and infant mortality communication with health facilities, including emergency transportation. This led to a 27% increase in facility-based delivery [25].

Several thematic reviews on mHealth projects and their overall impact on public health outcomes reveal that while mHealth interventions may positively influence CHW performances in low- and middle-income countries through iCCM to sick children with diarrhea, pneumonia and malaria, very few studies demonstrate impact on clinical outcomes [26]. Other systematic review analyses on the use of mobile mHealth tools by CHWs in the area of maternal and child health [27], HIV/AIDS and sexual and reproductive health provides some evidence on the importance of mHealth in improving quality of care, service efficiency and increasing capacity for program monitoring [28].

In 2013, the World Health Organization (WHO), under the Rapid Access and Expansion (RAcE) iCCM program, funded World Vision Canada and the Nigerien Ministère de Santé Publique (hereafter referred to as the Ministry of Public Health or MPH) to implement iCCM delivered through CHWs in four districts of southwestern Niger. The RAcE program supported MPH to introduce iCCM-delivered services through trained volunteers, known as *Relais Communautaire* or RCom. RCom differ from the regular MPH CHW program in Niger, in which the paid Community Health Agents (*Agents de Santé Communautaire* or ASC) are based at Health Posts and trained to a higher level. The iCCM approach used in Niger also focused on supervision – through inventory management, data collection review, clinical mentoring and problem solving – and a delivery approach, which included monthly community and health facility meetings.

An mHealth intervention was introduced among a sub-set of RCom. Samsung smartphones, equipped with a dedicated application, training and regular supervision were provided to 100 RCom. The mHealth intervention complemented the standard use by all RCom of advanced yet appropriate therapeutic tools, including artemisinin-based combination therapy (ACT), rapid diagnostic tests for malaria, amoxicillin, paracetamol, oral rehydration salts (ORS), zinc supplementation and referral to a higher level of care as needed. The value of mobile technology in delivering iCCM services by the RCom was assessed. The objective was to contribute to the body of quality research on mHealth in iCCM and inform and influence iCCM program implementation and policy by MPH Niger and WHO. The study took place in rural communities in two administrative regions of southwestern Niger, Dosso and Doutchi, located 120 and 150 km east of the capital city, Niamey and bordering the upper northwest corner of Nigeria (**Figure 1**); all as part of the larger iCCM RAcE project in four regions of Niger with approximately 1200 RCom in total.

**Figure 1:**
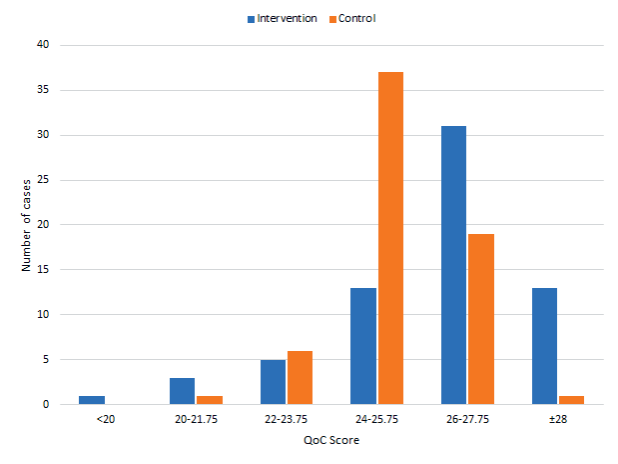
Maps of Africa (GoogleMaps), Niger and Dosso and Doutchi Departments showing location of study participants (RCom (*Relais Communautaire*) and children).

The primary research question focused on whether the use of a specially equipped smartphone (or mHealth intervention) could make a difference in the diagnosis and treatment by RCom of malaria, pneumonia and diarrhea in children aged 2 to 59 months. In addition, we assessed the effects of mHealth on RCom motivation and retention.

## METHODS

### Sampling

This was a controlled randomized cluster trial with two arms, taking into consideration an intra-group coefficient of 0.1, a significance level of 5%, a power of 80% and a cluster size of four (or four patients seen by each RCom). The Hayes and Bennett sample size calculation formula [29] was used to calculate the sample size: 252 patients/children in 63 groups (where one group is one RCom). The same sample size was also allocated to 63 RCom not using smartphones. By applying a dropout rate of 10%, a final sample of 554 patients needed for this study (or 227 in each of the intervention and control groups) was obtained.

A total of 130 RCom from 129 villages were selected randomly for the study: 66 in the intervention group (mHealth; out of a total of 95) and 64 in the control group (paper based; out of a total of 463). Ultimately, 65 community health centres were involved with 32 in Dosso District and 33 in Doutchi District. These two districts, both highly rural and about 120 to 150 km from the capital city, Niamey, and on the very northwest corner of Nigeria, were chosen from within the overall larger World Vision RCom training project for their closer proximity to Niamey and for their better security situation. The intervention group RCom had used the smart phone application for about six months prior to the beginning of the study. See **Table 1** for a socio-demographic description of the RCom.

**Table 1:** RCom (*Relais Communautaire*) socio-demographic and work context characteristics (Dosso and Doutchi Districts combined)

Characteristic	Intervention group		Control group	
**Number (n = 66)**	**Proportion (%)**	**Number (n = 64)**	**Proportion (%)**
**Age (years):**				
<20	2	3.0	2	3.1
20-29	9	13.6	17	26.6
30-39	27	40.9	20	31.3
40-49	23	34.8	16	25.0
>50	5	7.6	9	14.1
**Sex:**				
Male	54	81.8	35	54.7
Female	12	18.2	29	45.3
**Education:**				
Some primary	11	16.7	15	23.4
Primary	11	16.7	15	23.4
Some middle	41	62.1	32	50.0
Middle	2	3.0	1	1.6
Some high school	1	1.5	0	0.0
More than high school	0	0.0	1	1.6
**Marital status:**				
Married	64	97.0	60	93.8
Single	2	3.0	4	6.3
**Distance from health centre (km):**				
1-5	7	10.6	4	6.3
6-10	12	18.2	16	25.0
11-15	12	18.2	9	14.1
16-20	5	7.6	4	6.3
21-25	6	9.1	3	4.7
26-30	7	10.6	3	4.7
>30	17	25.8	25	39.1
**Number of supervisory visits:**				
0	21	31.8	10	15.6
1 or 2	18	27.3	23	35.9
3 or more	27	40.9	31	48.4

All RCom, including those in the intervention and control groups, received ten days of standard MPH iCCM training in their local languages (Hausa mainly in Doutchi and Zarma in Dosso). They were then deployed in their respective communities to diagnose and treat uncomplicated cases of malaria, pneumonia and diarrhea and refer children with severe illnesses. The intervention group RCom were provided Samsung smartphones loaded with a contextualized French version of the CommCare application (Dimagi Inc, Cambridge, MA, USA) which World Vision had prepared for multi-country use [30]. All RCom interactions with this research and all their work were in local languages Hausa and Zarma and they all had a minimum level of competency in French.

The smartphone/application-supported RCom enabled them to follow set diagnosis and treatment protocols during their encounter with the caregiver and sick child and it also contained a module for the control of drugs and supplies. All intervention RCom were trained for five days on the use of the smartphone and application by the local senior research staff.

A team with a trained clinician and assistant per district (Medical Doctor/Nurse; Medical Doctor/Master of Public Health) visited each RCom during normal service hours to assess QoC and levels of motivation and retention. They were trained for five days under the supervision of two MPH trainers with the support of the MPH Directorate of Statistics. Specifically-designed survey tools included RCom observational assessments and questionnaire surveys, clinical reassessments of the sick children, and questions designed to assess learning levels based on four presented case studies on diarrhea, cough, fever and referral. All 130 RCom were observed managing four children each, followed by re-examination by the clinicians. A total of 520 presenting sick children (aged 2 to 59 months) were diagnosed and treated by the RCom (264 intervention group and 256 control group).

The children were selected on a first-come basis on the days that the trained clinicians presented themselves in the communities with the RCom. Informed consent was obtained from the children’s caregivers. Ethical clearance was obtained from Ryerson University, Toronto, Canada and MPH Niger.

### Quality of care definition and measurement

Three dependent variables were measured: QoC provided by RCom, RCom motivation and retention. Quality assurance was achieved by a random audit of 20% of the overall sample. Using SPSS software (IBM Corporation, Armonk NY, USA), univariate and multivariate logistic regression analyses were performed to determine the relationship between the three variables and the potential explanatory effect of using CommCare-equipped smartphones in the context of community-based primary health care.

Since no standard measure of QoC for this purpose existed, a composite overall QoC score was developed specific to the diagnosis and treatment job responsibilities of the RCom. It included aspects of health screening, danger sign identification, and treatment which included referral, medication use and advising caregivers.

The QoC score was developed such that a 100% score could be achieved for each child so as to consider illness identification and delivery of care equally important to ruling out illness and avoiding unnecessary treatment or referral. A perfect score was achieved when the RCom asked all ten health screening questions (including mid-upper arm circumference, or MUAC) and correctly classified (ie, present or absent) the four major danger signs and six additional serious symptoms, made an immediate referral according to the presence of the four major danger signs and/or red MUAC, administered medication appropriately, and provided advice to caregivers. Scoring non-events was done to avoid distortion of results when there was no evidence of serious illness or need for medication.

The four referable danger signs included: convulsions; incapacity to drink or eat; vomiting; and lethargy or unconsciousness. A fifth serious danger sign was severe acute malnutrition, assessed by MUAC tape showing red, although this was not considered a referral condition in Niger. The six additional serious conditions were: chest indrawing; swelling of feet; fast breathing; diarrhea; blood in stool; and fever. These comprised the 11 conditions checked for by the RCom.

The maximum QoC score was 31 points; the score was weighted with two-thirds of the score allocated to screening questions and correct identification of the danger signs and signs/symptoms of serious conditions (**Table 2**).

**Table 2:** Components and criteria of the quality of care score

Component	Description	Score Criteria	Score
**Assessment**			
Health screening	RCom are expected to complete a full health screen which consists of 10 questions; one point was awarded for each question asked. The health screen addresses respiratory, gastro-intestinal, neurological, and systemic conditions including fever, swelling, cough, vomiting, etc.	1 point for every question asked	10
Identification of danger signs and other serious conditions/symptoms	Based on the response to health screening questions, RCom are expected to further assess, looking for four danger signs for immediate referral and seven other serious health conditions including red MUAC. One point was awarded for each of the 11 possible signs that were correctly confirmed by the clinician; the point was awarded whether the flag was correctly identified as positive (being present) or negative (not present).	1 point for every sign correctly identified	11
**Treatment**			
Referral	RCom are expected to refer sick children to a health centre when a child presented with at least one major danger sign (convulsions, lethargy/unconsciousness, feeding/drinking incapacity and vomiting).	3 points were awarded when a correct referral or non-referral occurred.	3
Medication administration	RCom are expected to administer four types of medications when appropriate. RCom are able to administer: ORS/Zinc for gastrointestinal /, ACT for malarial symptoms, Paracetemol for fever, and Amoxicillin for respiratory illness/pneumonia.	One point was awarded for each of the 4 medications that were administered or not administered correctly.	4
	The criteria for administering a medication were based on positive symptoms, rather than positive danger signs. Children presenting with diarrhea required ORS/zinc; with fever, they required Paracetemol; with vomiting or diarrhea combined with a fever, they required ACT; and with cough combined with a fever, they required Amoxicillin. Children that were referred to a health clinic were considered to have been treated appropriately regardless of whether medication had been administered or not.		
Advice given	RCom are expected to advise caregivers regarding the need for referral. If a referral occurs, for those returning home, one point was awarded for advice when it was given in each of the following three topics: home care, immunizations, and follow up.	3 points awarded if reason for referral was made; or,	3
		1 point awarded for each of the following: advice on home care, immunizations, and follow-up	
**Total score**			31

ACT – artemisinin-based combination therapy, ORS – oral rehydration solution, RCom – *Relais Communautaire*

A QoC score was first computed for each child; the mean score for the four children seen by each RCom (the cluster) was then used for comparison to determine whether the mHealth intervention was influential. An independent-samples two-tailed test using bootstrap to normalize the data was used to compare QoC scores for intervention and comparison groups. Statistical tests were completed using SPSS v.22.0 (IBM Corp, Armonk NY, USA). For further comparison between experimental groups, a Chi Square test was used, with calculations completed in Excel (Microsoft Corporation, Redmond WA, USA). An alpha level of 0.05 was used for all statistical testing as the minimum level of significance.

## RESULTS

There were no significant demographic differences between intervention and control groups at baseline, except that the intervention group was comprised of fewer female RCom (**Table 1**). A total of 520 sick children (264 intervention group and 256 control group) presenting to the RCom were diagnosed and treated. Overall, the symptom profiles of the children presenting were quite similar between the intervention and control groups with about 45% presenting with cough, 10% with diarrhea, and very few with vomiting. Approximately 70% of the children assessed showed signs of a fever with 40 to 50% of these children showing only signs of a fever with no co-existing cough, diarrhea or vomiting, depending on the group. Statistical comparison showed no significant difference in the mix between experimental groups based on the above symptoms (*P* = 0.10).

Small differences emerged between experimental groups in relation to signs and symptoms of serious illness and complexity. No significant difference between groups was seen for children diagnosed with the four immediately referable danger signs and red MUAC (*P* = 0.49); but a difference emerged between groups when all the danger signs were considered including referral signs (*P* = 0.000; data not shown). The differences in the latter were seen for fast breathing (73% vs 69% for intervention and control group, respectively), chest indrawing (47% vs 56%, respectively) and fever (9.3% vs 12.5%, respectively).

Small differences between groups were seen for case complexity. The experimental group had approximately 10% more cases presenting with multiple symptoms; however, the control group tended towards more complex, serious cases: 43% vs 45% cases had at least 1 of the 11 signs of serious illness, respectively; and 15% vs 17% for two or more of these signs.

### Quality of care

**Figure 2:**
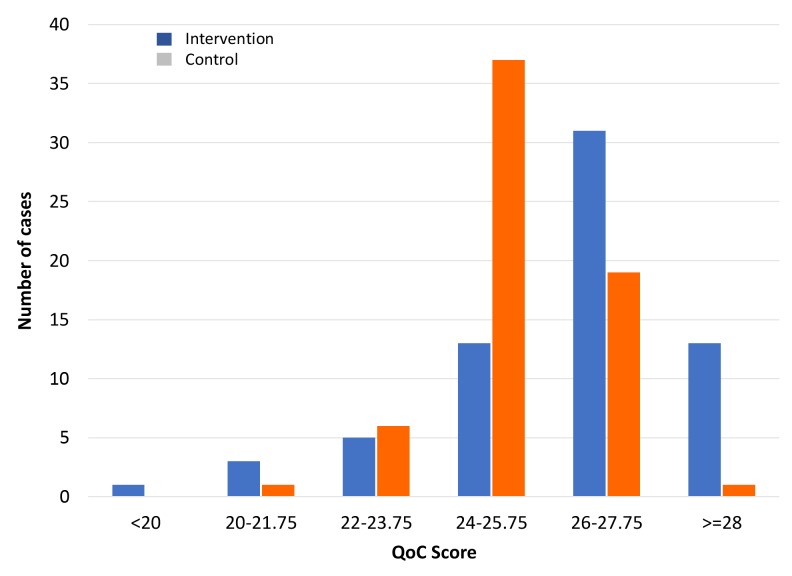
Distribution of quality of care score by study group (Intervention n = 66, Control n = 64).

The average QoC score for all 130 RCom was 25.77, SD 1.83, (or 83.1%), indicating satisfactory QoC overall. Identification and classification of danger signs and serious conditions/symptoms, medication administration and providing advice were all done well. The QoC per RCom was significantly higher for the intervention group than the control group (*P* = 0.009) indicating that use of the smart phone and mHealth application improved QoC, and this was attributable to better assessment. The effect, though, was small with a mean difference of 0.9 points (or 3.6% higher; *P* < 0.01) (**Table 3**). However, upon further analysis, 83.3% of the intervention group had a QoC score greater than 80% (25 out of 31), whereas only 67.2% of the control group had a score of 80% or higher (**Figure 2**), thus demonstrating that the average QoC values somewhat understate the intervention group’s effectiveness. QoC was calculated for each RCom by using the data collected for each of the four children seen (a cluster analysis). Individual per child scores were also compared and were very similar to the cluster calculation.

**Table 3:** Mean (standard deviation) quality of care score per RCom for key outcomes

	Intervention group	Control group
	**26.2 (2.1)**	**25.3 (1.4)**
Health screen*	7.4 (1.6)	6.4 (1.2)
Danger signs	10.5 (0.5)	10.6 (0.4)
Treatment:	8.3 (1.0)	8.4 (0.8)
-Referral	2.7 (0.6)	2.8 (0.4)
-Medications	3.1 (0.5)	3.0 (0.4)
-Advice	2.8 (0.4)	2.6 (0.5)

RCom – *Relais Communautaire*

*Significant difference between intervention and control arms, *P* < 0.001.

### Clinical assessment (screening)

**Figure 3:**
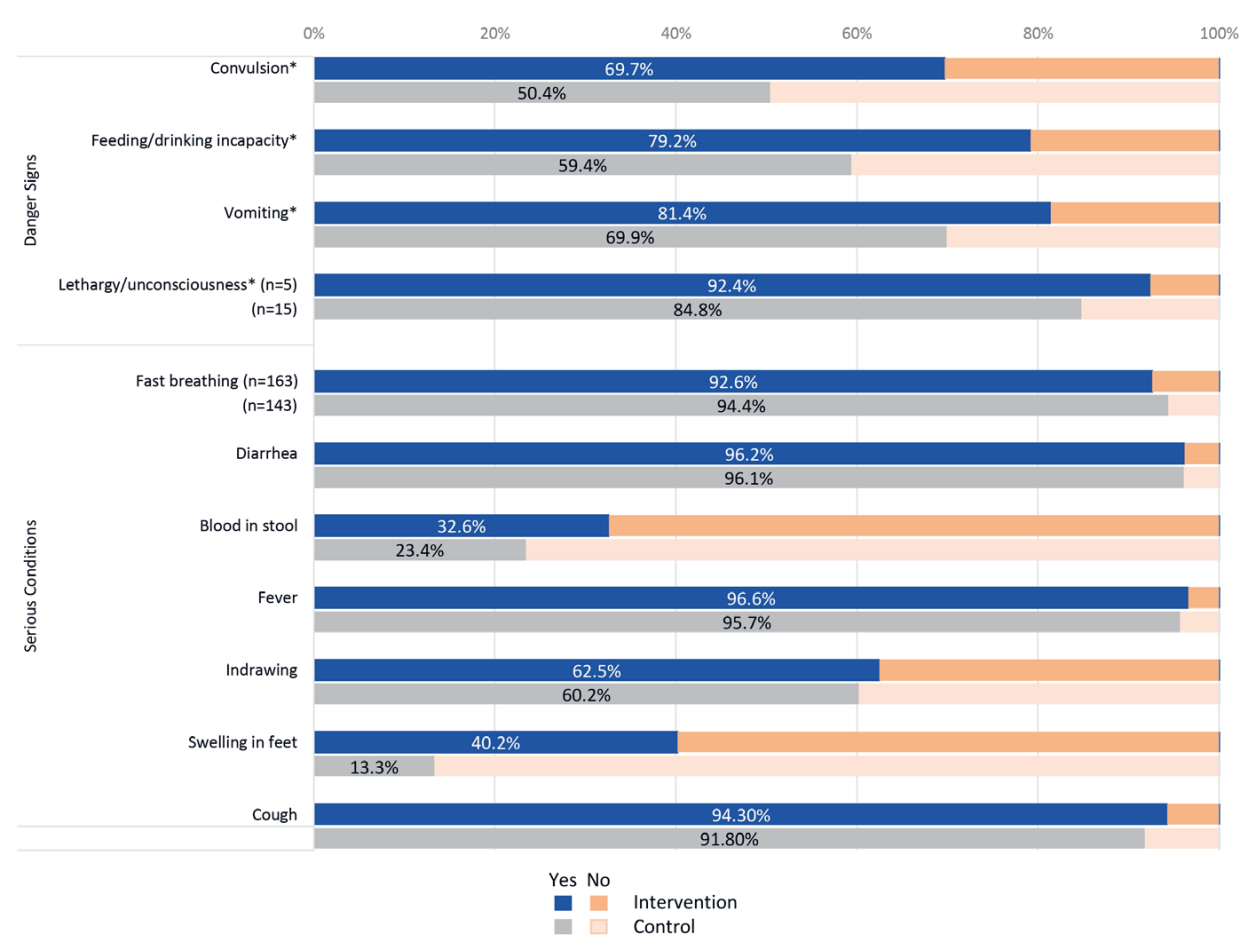
Percentage of cases examined by RCom (*Relais Communautaire*) for all danger signs and conditions, by study group (Intervention n = 264; Control n = 256). Asterisk – *P* < 0.010 for difference between study arms.

Analysis of various components comprising the QoC score showed the difference between the two study groups was driven by differences in health assessment (ie, screening), with the intervention RCom consistently asking one more question on average than the control group. The mHealth-enabled RCom trended towards a more complete health assessment with a higher proportion asking each of the ten health screening questions (**Figure 3**). This difference was significant (*P* < 0.01) for all four major danger signs (convulsions, incapacity of feeding, vomiting, and lethargy/unconsciousness) meaning that a significantly greater proportion of children were assessed for these important referable factors among RCom with the mobile phones (data not shown).

The intervention group was also significantly more likely (40.2% vs 13.3%, *P* < 0.01) to look for swelling of the feet (nutritional edema) and more likely (non-significant) to assess for blood in stool (data not shown). Conversely, the control group was significantly more likely to assess for severe acute malnutrition using MUAC tape measurement showing red (*P* < 0.05). Cough and diarrhea were almost always equally assessed and the control group had considerably more cases with fever (73 vs 51 cases).

### Classification (diagnosis)

The QoC subscores for correct classification of all 11 referable danger signs (4) and serious conditions (7) were similar between the intervention (10.5, SD 0.5) and control groups (10.6, SD 0.4) (**Table 3**). Of the four main danger signs, there was no significant difference in correct classification between groups (**Table 4**). More generally, the intervention group had a lower rate of missing a danger sign compared to the control group (29.2% vs 32.3%) but a higher rate of being incorrect (56.5% vs 28.3%). The most frequently missed signs across both groups were chest indrawing (54.5%), fever greater than 7 days (20.5%), and fast breathing (19.3%). All RCom generally used RDT well, but answered assessment questions related to the four case studies poorly.

**Table 4:** Proportion of RCom classifications for general danger signs which corresponded to clinician observer’s classifications by study group

	Intervention (n = 264)				Control (n = 256)				*P*-value comparing intervention with control
	**% correct**		**% missed**	**% incorrect**	**% Correct**		**% missed**	**% incorrect**	
	**Positive**	**Negative**			**Positive**	**Negative**			
**Danger signs:**									
Convulsions	0.0	99.2	0.0	0.8	0.0	100.0	0.0	0.0	NS
Incapacity of feeding	0.4	97.7	0.8	1.1	0.4	98.4	0.4	0.8	NS
Vomiting	1.1	98.5	0.4	0.0	0.0	98.8	0.8	0.4	NS
Lethargy	0.4	98.1	0.4	1.1	0.0	98.8	0.0	1.2	NS
Other conditions:									
Fast breathing	25.4	60.6	4.5	9.5	26.6	62.9	3.5	7.0	NS
Diarrhea >14 d	0.4	97.7	0.0	1.9	0.4	98.8	0.0	0.8	NS
Blood in stool	0.4	96.6	0.4	2.7	0.4	99.2	0.0	0.4	0.021
Fever >7 days	0.4	91.3	3.4	4.9	2.0	93.0	3.5	1.6	NS
Chest indrawing	12.5	72.0	6.8	8.7	12.9	71.1	11.7	4.3	0.027
Swelling of feet	0.4	97.0	0.4	2.3	0.0	99.2	0.4	0.4	NS
Red MUAC	3.0	95.8	0.4	0.8	0.4	98.0	1.2	0.4	NS

RCom – *Relais Communautaire*, MUAC – mid-upper arm circumference

The control group was significantly more likely to correctly classify chest indrawing (*P* = 0.021) and blood in the stool (*P* = 0.027) (**Table 4**). Otherwise, there was no significant difference in the identification and classification of conditions between groups of RCom, including diarrhea and fever (*P* > 0.05 for both).

However, difficulty for RCom to correctly identify the danger signs and serious conditions was not apparent in the QoC score since the average sub-score for identifying danger signs was 10.55 out of 11, or 96%, for the whole group. When this performance was reviewed more closely, it was apparent that the assessment score was strongly influenced by the ability to recognize when a danger sign was absent (**Table 4**). This effect was not surprising given that 58% and 56% of the children seen in the intervention and control groups, respectively, had no danger signs or other serious conditions or symptoms of illness.

### Treatment and counselling

**Figure 4:**
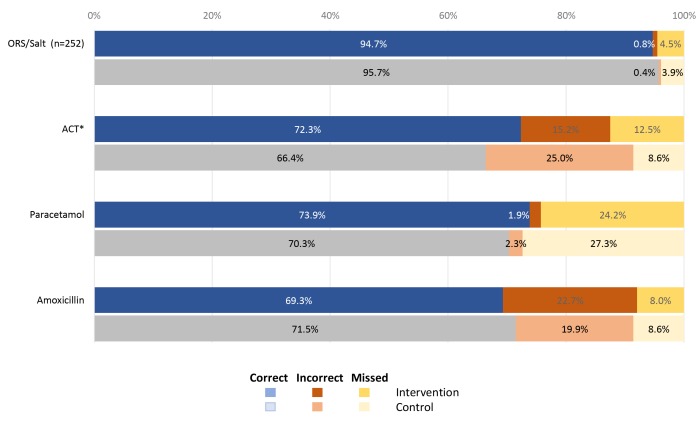
Correct RCom (*Relais Communautaire*) administration of medication to each case, by study group (Intervention n = 264; Control n = 256). Asterisk – *P* = 0.023 for difference between study arms.

The QoC subscores for treatment were similar between the intervention (8.3, SD 1.0) and the control group (8.4, SD 0.8) (**Table 3****)**. However, a significant difference emerged between the intervention and control groups with the intervention group more correctly administering ACT as indicated (72.3%) compared to the control group (66.4%) (*P* = 0.012), including being less likely to administer the drug when it was not required (**Figure 4**).

Among the most important conditions, RCom in the control group had significantly fewer errors when treating fast breathing (*P* = 0.022) (**Table 4**) and were significantly better at giving advice about the use of either the antibiotic, ORS, zinc or anti-malarial (*P* < 0.05). There was no significant difference between groups in the use of amoxicillin to treat pneumonia (cough and fast breathing) which together with good RDT use contributed to good overall QoC in both groups. Regarding other conditions or symptoms, the control RCom had significantly fewer errors when addressing cases of blood in the stool (*P* = 0.035).

Medications were administered similarly by all RCom (**Figure 4**); however, of concern was the relatively high number of incorrect and missed administrations of medication. ACT treatment was missed or prescribed incorrectly in 27.9% of cases by intervention RCom and 33.6% of cases by control group RCom, with similar numbers also seen for paracetamol (26.1% and 29.7%) and amoxicillin (30.6% and 28.5%). Use of drugs (correct diagnosis and prescription) was appropriate, therefore, about 70% of the time.

The proportion of caregivers who were counselled by the RCom to give more fluids and continue to feed their child in cases of diarrhea was significantly higher in the intervention group (64.9% vs 46.0%, *P* = 0.049). On the other hand, intervention group RCom gave significantly less advice to mothers/caregivers on the dosage of medication, (*P* = 0.028), but were somewhat more likely to have caregivers properly explain back drug dose and administration on site.

There was little to no difference in caregiver satisfaction with the RCom’s work and the willingness of caregivers to return for more services in the future, which were all generally high.

### Referrals

Within the QoC calculation there was no difference between the RCom groups with respect to referral, when considering correct, missed and wrong referrals (*P* = 0.33). Both did relatively well, scoring just over 90% correct, though mainly on the strength of not referring when not needed. The mHealth-enabled RCom, however, did significantly better (*P* = 0.012) in referring to a higher level of care when needed, ie, when danger signs were present. Using a restricted sample of referrals (referrals confirmed needed and referrals made), statistical differences emerged based on correct, missed and wrong referrals (*P* = 0.044): the intervention group was more likely to correctly refer (11/13 vs 2/7 for intervention and control groups, respectively), and less likely to miss the referral (2/13 vs 5/7). Furthermore, the intervention group was less likely to be wrong (ie, make referral when a referral was not required) (21/32 vs 15/17 for intervention and control groups, respectively).

To better understand these referral results, a similar comparison of referrals was completed based on the RCom identifying a referral sign and making the referral; this analysis showed no significant difference in results between groups (*P* = 0.240) thus suggesting that missed referral signs were the main reason. Surprisingly, wrong referrals (20/32 vs 14/17 for intervention and control groups, respectively) suggests that RCom based their decision to refer on other criteria in addition to the established referral criteria.

Incorrect referrals, where the clinician did not agree with the RComs’ decisions to refer, were not a large problem, as only 36 such referrals occurred (21 and 15 in intervention and control, respectively).

### Supervision, retention and motivation

**Figure 5:**
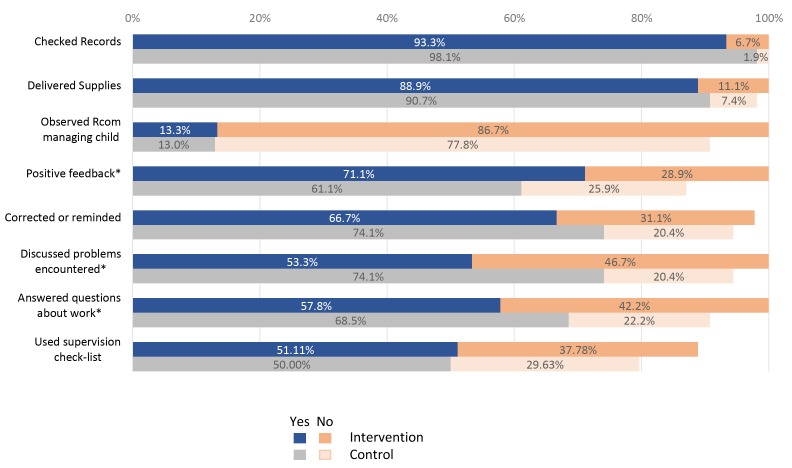
Proportion of RCom (*Relais Communautaire*) whose supervisor performed each duty during visit, by study group (Intervention n = 45; Control n = 54). Asterisk – *P* < 0.050 for difference between study arms.

RCom in the control group received more supervision in terms of numbers of supervisory visits (**Table 1**), including complete lack of supervision in the last three months at about half the rate (15.6% vs 31.8%) as well as a somewhat better quality of supervision as reflected by supervisor actions (**Figure 5****)**. In general, regardless of smart phone use, RCom located less than 15 km from their supervisor provided better QoC compared to those located more than 15 km from their supervisor (data not shown).

RCom in the intervention group reported being slightly less likely to be retained compared to RCom in the control group (21.2% vs 17.2%) and reported poor working conditions more often (24.2% vs 15.6%). RCom retention also depended on education level across both groups: those at the secondary level (first cycle not completed) were 13.9 times more likely to be retained compared to those with a primary level not completed. Both groups were equally satisfied with their work environment (60% positive, with 20% non-response rate).

There were no statistically significant differences between both groups of RCom in their answers to 15 questions on motivational factors; though with the direct question about job satisfaction, RCom in the intervention group were slightly more satisfied than those of the control group (93.9% vs 85.9%; *P* = 0.256). Almost all RCom reported feeling respected, proud of their work and committed to it. The distance separating the RCom from her or his supervisor constituted a major factor in RCom motivation. RCom who were located less than 15 km from their supervisor were more motivated than those located more than 15 km from their supervisor. Academic training levels also had an effect on RCom motivation regardless of mHealth group assignment; those who completed primary school level were 3.6 times more likely to be motivated compared to those who had not completed primary education.

A summary overview of all the key study findings is found in **Table 5**, indicating where the mHealth intervention had a positive effect, but also several instances where the control group RCom did better than the mHealth intervention group.

**Table 5:** Summary of key findings of differences when comparing RCom intervention and control groups

Level of differences	Summary of findings
1. Statistically significant findings in favour of mHealth equipped RCom	• Greater proportion of children examined for the four major danger signs by the RCom
	• Greater proportion of children examined for swelling of both feet by the RCom amongst additional danger signs
	• Greater proportion of children with diarrhea whose caretakers got advice to give more fluids and to continue feeding
	• Greater proportion of children in need of an antibiotic, ORS, zinc and/or anti-malarial whose caregivers received at least one advice about drug dosage and administration
	• Greater proportion of RCom whose supervisor provided positive feedback about doing a good job
	• More suggested that World Vision can increase RCom job satisfaction by providing a salary and financial support (as they find their RCom work difficult to balance with the need to have income generating work)
	• More showed less appreciation of motivation statements
	• More suggested that cell phones could be used for other functions, like calculator and timer
2. Not statistically significant findings but which showed a higher positive difference in favour of mHealth equipped RCom	• Greater proportion of children examined for cough and diarrhea by the RCom
	• Greater proportion of children correctly referred for the all danger signs/classified diseases by the RCom
	• Greater proportion of children in need of antibiotic, ORS, zinc and/or anti-malarial who received the first dose of the treatment right away
	• Greater proportion of children whose breathing rate had been evaluated and compared favourably within a gap of ±3 between measures by RCom and clinicians
	• Proportion of children given an antibiotic, ORS, zinc and/or anti-malarial whose caregiver could explain how to administer the treatment (further complementing the statistically significant finding above)
	• Received fewer supervisory visits in the last three months than the control group
	• Had a more negative perception of their working conditions (worse than control)
	• Suggested that World Vision could increase their job satisfaction by working to improve transportation (complementing that of financial resources above)
3. Statistically significant findings in favour of the RCom control group, those not equipped with the mHealth intervention	• Greater proportion of children assessed for severe acute malnutrition by MUAC tape colour reading by the RCom (as one of the other danger signs)
	• Greater proportion of children whose classifications given by the RCom corresponded to the clinicians’ in two major areas (diarrhea with blood in stool and fast breathing)
	• Greater proportion whose supervisor discussed problems and answered questions during the most recent visit
	• More control RCom were women
4. Not statistically significant findings but which showed a high positive difference in favour of the RCom control group not using the mHealth intervention	• Greater proportion of children with confirmed fever and positive RDT who received an anti-malarial from the RCom
	• Greater proportion of children with fever confirmed by high temperature who received Paracetamol from the RCom
	• Greater number of times the RCom received a supervisory visit in the last 3 months; the control group RCom received more supervisory visits overall
	• Greater proportion of RCom whose supervisor corrected or reminded them of things during the most recent visit
	• More satisfaction with their work environment
	• Have a better perception of their working conditions
	• More suggested that World Vision can increase their job satisfaction by providing them materials, medications and food support
5. No difference between RCom groups	• Proportion of children examined for fever
	• Proportion of children whose breathing rate had been evaluated and compared favourably within a gap of ±3 between measures by RCom and clinicians
	• General treatment of children
	• Proportion of children with cough and fast breathing who were prescribed Amoxicillin by the RCom
	• Correct classifications (as verified by the clinicians) for: diarrhea less than 14 days and no blood in the stool; diarrhea for 14 days or more; blood in the stool; fever for last 7 days; fever for less than 7 days; and chest indrawing
	• Caregiver satisfaction of the RCom’s work and their willingness to return for more services in the future (but was all generally high)
	• Most poorly answered the questions related to the four case studies.

RCom – *Relais Communautaire*

## DISCUSSION

The QoC delivered by RCom was dependent on their ability to complete a full screening assessment, accurately assess the child’s health status and deliver appropriate care, whether administering medication, advising on home care and follow up, and referral to a nearby health centre if appropriate. Overall, QoC by RCom was high, with the intervention group having a small but significant better score (3.4%). This small difference appears to be related to the health screening process, with the mHealth-enabled group asking one more question on average than the control group, suggesting slightly improved protocol adherence in this area attributable to mHealth use.

Irrespective of the considered general danger signs, the proportion of children examined for danger signs was significantly higher among the RCom who used the CommCare equipped smartphone than those who did not. This could be attributed to the intervention RCom being more attentive and following procedure when looking for general danger signs than the control group RCom. The higher proportion of children presenting with multiple symptoms to the intervention group may have biased them towards a better assessment, shifting their QoC score upwards.

Most RCom performed well in correctly identifying the presence or absence of danger signs, with the most frequently missed serious conditions being chest indrawing, fast breathing and fever. The intervention group was more likely to incorrectly identify chest indrawing; while the control group was more likely to miss this sign. RCom also failed to recognize threshold values; for example, pulse was assessed correctly, but not assigned as a condition even when it exceeded the threshold. Additionally, results showed that the three screening questions indicating systemic involvement were not being asked; the extent of screening for illness severity appeared to be limited. The intervention group missed classifying (ie, diagnosing) a much smaller percentage of the four main iCCM danger signs including red MUAC (30.8% vs 71.4%), with missing the danger signs attributed to inadequate screening or low skill level.

There was a relatively high number of incorrect and missed administrations of medications that, when combined, were greater than the number of correctly administered medications; this being of great concern and an area where supervision should be focused. The use of the mHealth application in correctly administering medications only had a significant effect for ACT medication with the intervention group prescribing it better and being less likely to prescribe when the drug was not required, a positive finding that might contribute to lessening the development of drug resistance. The availability and use by all of the RDT also contributed to ensuring a better QoC. In general, amongst all RCom, the correct use of drugs, including amoxicillin, was similar at about 70%, and over 90% for ORS.

Consideration should also be given to factors related to the child’s family. For example, a caregiver who has prior experience with sick children may delay seeking medical help or encourage the RCom to administer a medication they consider helpful. In our study, mHealth RCom showed better communication skill with caregivers, suggesting a potentially useful strategy to counter such influences on treatment and referral.

While referral in general was poor, intervention RCom did better, with much fewer missed referrals when needed compared to the control group (2/13 or 15% vs 5/7 or 71%, respectively), and fewer incorrect referrals (21/32 or 66% and 15/17 or 88%, respectively). Better referral performance in the mHealth group can potentially build community-wide confidence in the health system, so important in such remote and isolated settings. Amongst both groups, though, referrals were more likely to occur when the distance from a clinic was farther and RCom did not delay referral because of a long distance to further medical assistance. Since RCom in the control group tended to be further from a health clinic (**Table 1**), distance from a health centre may have masked an mHealth effect.

In general, the use of the CommCare mHealth application contributed to a slight improvement in QoC given to sick children by RCom. As can be deduced from the data, RCom in both groups performed well in their work, providing needed health services to children in their communities, though many areas were identified for improvement, including better training, continuing education and better supervision. RCom in the intervention group were slightly more satisfied and more likely not to be retained than those of the control group, which may have been related to sex (there were many more male RCom in the intervention group) and to using an mHealth-enabled smartphone in a village clinical setting, potentially having the perceived added community status and potential career mobility that it confers, possibly more so to males vs females. This potential set of impacts on social dynamics following mHealth introduction merits further investigation.

It should also be noted that carrying out such a study in one of the poorest areas of the world was a challenge, but constant progress was made and plans were mostly attained by the diligent staff. There was also good technical support through WHO. Accessing cloud services, having constant access to electricity, having reliable transport (mostly small motorcycles), distances, and lack of maps and travel landmarks were further challenges. Carrying out one of the first ever clinical randomized controlled trials on mHealth was a great learning experience by all; and progress, even meagre, was a commendable achievement and should continue. Seeds have been planted in the dry ground.

### Limitations

The scope of this study was focused on detecting differences in case management of children by CHWs due to use of a smartphone equipped with a special application. The findings have several limitations. First is the possibility that the initial and subsequent training on using the smartphone could have been better, along with closer and more specific supervision to use the technology to maximum effect to leverage effectiveness of the iCCM program. Subsequently, the results are applicable to settings where World Vision’s version of the CommCare application in support of iCCM programming is deployed. Our definition of QoC could have been faulty, yet it was composed of logical variables that were adequately measured. Finally, the study was not designed to assess efficiency gain in RCom work patterns or in the potential cost-benefit or cost-effectiveness of the mHealth intervention, nor was it designed to evaluate various other possible smartphone applications [13,15,17,18].

## CONCLUSIONS

In this study, RCom using the CommCare-provisioned mHealth application on a smartphone provided only somewhat better QoC for sick children, with the most pronounced difference being an improvement in asking about danger signs. They did better in screening for four major danger signs, were better at referring when needed, but received less supervision. By contrast, they were more difficult to retain and were more inclined to want financial reward for their work. The control group without mHealth support did better on several measures; however, by many other measured variables, there was little to no difference between the groups. There was no clinically relevant effect of mHealth, except for somewhat closer questioning regarding danger signs and better referral. Both groups were comparably happy and satisfied with their work and motivated to perform well, despite poor supervision.

The positive impact of mHealth on RCom diagnosis and treatment of the three most prominent childhood diseases, although encouraging, was relatively small and therefore the results of this study do not lead to a definitive recommendation to scale up mHealth in this setting, especially considering trade-offs related to associated costs and logistics. It would be essential to address identified gaps in the use of the mHealth intervention. While it is likely that the mHealth program could evolve into a more effective one, especially if supervision and training were enhanced, the cost-benefit remains poorly understood and the potential to improve overall RCom performance through other strategies, including better overall supervision, continuing education and building organization culture [31], should remain a priority for iCCM.

The RAcE project in Niger offered the opportunity to perform a rigorous study to help improve iCCM implementation and understand better the effects of mHealth on leveraging RCom/CHW services. The project assessed the tacit belief that implementation of information and communications technologies (ICT) to equip front-line health workers will improve health outcomes in children. Considering the way the mHealth intervention was implemented, the incumbent poverty of the study location and the cost and additional logistic requirements of mHealth implementation, this study cannot conclusively defend its implementation to improve RCom/CHW performance and health outcomes.

This study’s findings, showing very modest potential value of the mHealth application to support iCCM trained CHWs, prompts a re-examination of the role of mHealth in supporting such programs to deliver quality health outcomes to infants and young children. Our results, especially those which can be used to improve mHealth implementation and for scale-up, can help iCCM program managers address aspects of mHealth design and deployment that may make a difference in its value-add prospects in the future.
